# A Contemporary Analysis of 2500 Consecutive Fistulograms Performed in the Office Based Lab (OBL): Assessing Durability and Indications for Early Reintervention

**DOI:** 10.51894/001c.144146

**Published:** 2025-09-30

**Authors:** Robert Molnar

**Affiliations:** 1 Vascular Surgery Henry Ford Genesys; Michigan Vascular Center

## 45

### INTRODUCTION/BACKGROUND

Hemodialysis (HD) access maintenance is a vital component in maintaining effective HD. Most HD patients have ArterioVenous Fistulas (AVFs) or ArterioVenous Grafts (AVG) as their HD access circuit. HD access dysfunction leads to significant disruptions. Interventions are frequently required to maintain optimal function.

### AIMS/OBJECTIVES

To assess the characteristics and durability of fistulograms performed, we analyzed 2500 consecutive fistulograms performed exclusively at two high volume vascular surgeon OBLs.

### METHODS

A retrospective chart review of 2500 consecutive fistulograms at two OBLs was conducted. Fistulograms performed within 3 months of a previous fistulogram were further studied to determine the primary indication for intervention and the primary treatment location and whether the intervention was in the AVF/AVG (circuit), the central circulation, or both.

### RESULTS

Of the 2500 fistulograms, 678 (27%) were a first-time intervention, 1818 (73%) had had a previous fistulogram within the three-month period reviewed. 876 (35%) of the 2500 had a previous fistulogram within 3 months and are considered early reinterventions. Of the 35% early reintervention procedures performed, the most frequent indications were stenosis by duplex (44.75%), high venous pressure (43.38%) and prolonged bleeding (34%). The remaining indications were 15% or less each and included extremity swelling, difficult cannulation, poor clearances, clotted access and failure to mature. The principal target lesion was in the AVF/AVG (circuit) only 273 (31%), in the central circulation only 188 (21%), in both regions with the central lesion being the primary target 258 (29%) and in both with the circuit lesion being the primary target 143 (16%).

### DISCUSSION/CONCLUSIONS

Early reintervention HD access fistulograms are common and represent a substantial subset (35%) of the 2500 consecutive fistulograms reviewed. The most common indications for intervention were stenosis by duplex, high venous pressure and prolonged bleeding. The most common target lesion was identified in the HD access circuit only in 31%, however approximately 20% had isolated central circulation pathology and 45% had lesions both in the circuit and the central circulation. Further prospective study is required to identify optimal treatment strategies to avoid early reintervention in the performance of HD access maintenance.

